# Eating for Efficiency

**Published:** 1915-05

**Authors:** Manton M. Carrick


### Eating for Efficiency.

BY MANTON M. CARRICK, M. D.

It is no longer a fashion for health faddists to urge the ascetic alternative suggested by the ancient axiom, "Would you live to eat, or eat to live?" As a matter of fact, we eat for efficiency, though many of us lose sight of this important fact in the quality, and quantity of our food, to say nothing of the matter in which we dispose of it. Food bears exactly the same relationship to the body as coal to the engine, only more so, as the human body absorbs food and converts it into vital energy. A good fireman sees that his engine is fired with coal possessing a reasonable amount of heat-producing properties. Is it not, therefore, quite as important that the human body shall have its source of energy properly adjusted to its needs?

Most people injure their health and shorten their lives by not giving themselves time to enjoy their food. This bolting habit is, I believe, the most pernicious one when it comes to a question of health, for how much actual good can ever be derived from food that is only partially masticated, and never tasted at all? No, this is not a Fletcher dissertation, nor is it from the viewpoint of a faddist. It is just a plain, common-sense way of looking a practical everyday subject in the face.

"Men are animals, and as such they have a right to enjoy without reproach the pleasures of animal existence which maintain health, strength and life itself." Thus wrote no less a personage than President Eliot of Harvard many years ago.

The great menace to life is not gluttony, but bolting. By eating in a more leisurely fashion, much more pleasure and good can be gotten out of one mouthful of steak than from a whole platterful that is bolted.

Savarin, the Frenchman, once said that the destiny of nations depends how and what is eaten. Nor is this sweeping statement an exaggeration.

Remember, there are two doors to the mouth cavity—the front door which admits the food, and the back door that releases it. Taste is the keeper of both portals. So the first pledge I would extract from you is that you do more tasting. Swallow nothing that has not been reduced to a state of complete liquefaction. You will live longer, be happier while you do live, and make other people happier if your digestion is normal.

May I not add a friendly warning against fried foods. Frying coats the outside of the food with a layer of fat not easily penetrated by the digestive juice. For this reason fried foods should receive even more attention from the standpoint of thoroughly masticating. Roasted and broiled foods are always better, and more easily digested.

Violent exercise immediately after meals is not desirable, as vitality is thus taken away from the muscles of the stomach in their effort to aid digestion.

And remember that no amount of tea, coffee or any other beverage will add to the living tissue of the system. A cup of coffee is merely a goad to tired nerves and tissues, just as some men use a whip to a broken-down old horse. As to water, none of us partake of this beverage freely enough, and if we replenished the liquids of the body with pure water, instead of other beverages, we would be far better off.

Don't bolt! Taste! Drink plenty of *water!*

